# An Integrated Classification and Association Rule Technique for Early-Stage Diabetes Risk Prediction

**DOI:** 10.3390/healthcare10102070

**Published:** 2022-10-18

**Authors:** Doaa Sami Khafaga, Amal H. Alharbi, Israa Mohamed, Khalid M. Hosny

**Affiliations:** 1 Department of Computer Sciences, College of Computer and Information Sciences, Princess Nourah bint Abdulrahman University, P.O. Box 84428, Riyadh 11671, Saudi Arabia; 2 Faculty of Computers and Informatics, Zagazig University, Zagazig 44519, Egypt; 3 Faculty of Engineering and Computer Sciences, King Salman International University, Tor Sinai 46512, Egypt

**Keywords:** classification algorithms, early-stage diabetes, disease prediction, LOF, BBC, data mining, association rules and healthcare data analytics

## Abstract

The number of diabetic patients is increasing yearly worldwide, requiring the need for a quick intervention to help these people. Mortality rates are higher for diabetic patients with other serious health complications. Thus, early prediction for such diseases positively impacts healthcare quality and can prevent serious health complications later. This paper constructs an efficient prediction system for predicting diabetes in its early stage. The proposed system starts with a Local Outlier Factor (LOF)-based outlier detection technique to detect outlier data. A Balanced Bagging Classifier (BBC) technique is used to balance data distribution. Finally, integration between association rules and classification algorithms is used to develop a prediction model based on real data. Four classification algorithms were utilized in addition to an a priori algorithm that discovered relationships between various factors. The named algorithms are Artificial Neural Network (ANN), Decision Trees (DT), Support Vector Machines (SVM), and K Nearest Neighbor (KNN) for data classification. Results revealed that KNN provided the highest accuracy of 97.36% compared to the other applied algorithms. An a priori algorithm extracted association rules based on the Lift matrix. Four association rules from 12 attributes with the highest correlation and information gain scores relative to the class attribute were produced.

## 1. Introduction

Based on World Health Organization (WHO) reports [1,2], about 72% of global mortalities result from non-communicable diseases. Without intervention, this percentage will reach 90% by 2030 [3]. Diabetes is a set of non-communicable diseases that affects blood sugar (glucose). Despite the type of diabetes, the main cause is a sugar disorder in the blood, which usually results from the body’s inability to utilize insulin [4]. People with diabetes suffer from losing weight without intention, frequent nighty urination, and blurry vision. In the long run, diabetes can lead to serious health problems such as neuropathy, nephropathy, retinopathy, or cardiovascular disease [5].

Most people with diabetes live in low, or middle-income countries, and it is estimated that their number will increase from 85 million to 230 million by 2030 [6], which negatively impacts health care systems [7].

As a consequence of the increasing consciousness of diabetes risk, several studies have utilized machine learning algorithms for the early detection of diabetes [8,9].

Machine learning can be vital in predicting diseases and making critical health-related decisions based on an automatic learning process. Different classification and clustering algorithms have been widely used in this learning process. However, this process is greatly affected by data quality. For example, imbalanced datasets with many outliers greatly affect model results and accuracy.

The imbalanced dataset problem has been approached by many researchers [10,11]. Outlier detection algorithms have also been applied in previous studies [12,13]. Classification machine learning algorithms such as Support Vector Machine (SVM), Naïve Bayes (NB), and Decision Trees (DT) have been applied in healthcare systems to predict various diseases such as chronic kidney disease and heart disease [14]. Association rules have also been widely used as a mining technique for knowledge extraction [15,16].

Nevertheless, an integrative approach that combines classification algorithms with association rules for extracting knowledge and improving prediction model accuracy has not yet been introduced. Hence, we propose an integrative approach that utilizes Local Outlier Factor (LOF), and Balanced Bagging Classifier (BBC) classification algorithms, and association rules to predict diabetes early on. In addition, an a priori algorithm is utilized to find relationships among different risk factors. Applying the proposed approach is expected to reduce further health complications caused by diabetes due to early disease detection and early intervention. The dataset used in this study was collected using direct questionnaires from patients of the Sylhet Diabetes Hospital in Sylhet, Bangladesh.

The remainder of this paper is organized as follows. Section 2 summarizes related work. Section 3 presents the studied dataset along with the proposed approach. Section 4 discusses the results of a performance evaluation for the proposed approach. Section 5 concludes the paper and presents intended future research areas.

## 2. Related Work

Diabetes has been classified as one of the main mortality factors worldwide [17,18]. However, it has been shown that health status could be improved by careful diet and healthy exercise [19]. Hence, early diagnosis and prediction of diabetes is essential and could save lives. Machine learning has been widely used for disease prediction [20,21,22,23,24]. For example, Otunaiya and Muhammad [25] predicted chronic kidney disease using the Naïve Bayes (N.B.) and J48 decision tree. Their results showed that J48 outperformed the N.B. Yu et al. [26] applied a Support Vector Machine (SVM) to a dataset collected from the National Health and Nutrition Examination Survey to diagnose diabetes. Algorithm performance was measured using AUC, which achieved 83.5% for detecting diagnosed and undiagnosed diabetic patients. Ozcift and Gluten [27] applied a model based on 30 classifiers of heart diseases, Parkinson’s, and diabetes datasets. Their model outperformed other models in terms of accuracy. Chen et al. [28] applied an integration between K-Means and J48 decision trees in a PIMA Indian diabetes dataset. K-means was utilized as a data reduction technique, while J48 was the main classification algorithm. Their results showed a prediction accuracy of 90.04%. Several studies have considered neural networks (N.N.) as a powerful tool for disease prediction and diagnosis. For example, Saragih et al. [29] used an extreme learning machine to identify Jatropha Curcas disease. However, N.N. algorithms are highly dependent on learning rate, which is sometimes too low to find results in a reasonable time [30].

One of the most challenging problems in machine learning is dealing with outliers and managing imbalanced data sets. An outlier can be defined as a data point that differs significantly from other points in the dataset [31]. Outliers sometimes return to normal variability, which may indicate an experimental error. However, outliers can lead to serious inaccuracies in prediction models. Hence, outlier detection is a necessity for building a reliable prediction model. It has been shown that removing outliers from a dataset improves prediction model accuracy [32]. Outlier detection can be done through various methods. One of these methods is the Local Outlier Factor (LOF), a density-based method that uses Euclidian distance and k-Nearest Neighbour (kNN) to estimate local density [33]. The main strengths of LOF are identifying local density and determining local outliers. Several studies have shown promising results using LOF as an outlier detection technique.

Yan et al. [34] proposed a hybrid outlier detection technique based on the KNN algorithm and a Pruning based algorithm. Their results show that the proposed hybrid technique is superior to conventional KNN and LOF in efficiency and accuracy. Budiarto et al. [35] applied the K-Means algorithm, the LOF, and One-Class Support Vector Machine to two hospital drug use datasets. Their study showed that the One-Class Support Vector Machine algorithm outperformed the other algorithms in finding outliers.

Furthermore, the imbalanced dataset problem is considered a serious problem in machine learning and data science application models as it leads to unreliable and biased results. The problem happens when the number of data points in the majority and the minority classes differ significantly. Several studies have shown that handling the imbalanced dataset problem positively impacts classification and prediction models [36,37]. Imbalanced dataset problems can be handled by different techniques, such as the Balanced Bagging Classifier (BBC) [38]. Anbarasi and Janani [39] proposed a classifier ensemble technique based on a random forest algorithm to overcome inaccuracies associated with conventional classifiers. Their proposed multi-classifier techniques proved more accurate and efficient when applied to imbalanced healthcare datasets. Tuli et al. [40] proposed a novel approach called HealthFog, which integrates deep learning in Edge computing devices and then deploys it for automatic heart disease prediction and analysis. Their approach makes use of IOT and FogBus. According to their results, HealthFog achieved better quality of service in different fog computation scenarios.

It has been shown in several previous studies that model accuracy can be significantly increased if the outlier and imbalanced data problems can be solved. However, none of the previous studies has utilized the Local Outlier Factor with Balanced Bagging Classifier to handle outlier and imbalanced data problems, particularly for diabetes datasets.

This study proposes an integrative approach that utilizes the Local Outlier Factor (LOF), Balanced Bagging Classifier (BBC), classification algorithms, and association rules to extract knowledge to help predict diabetes early. The proposed approach is expected to improve prediction model accuracy and enable early detection of diabetes, which allows for early intervention and prevention of further health complications that may lead to death.

## 3. Methodology

**Figure 1:**
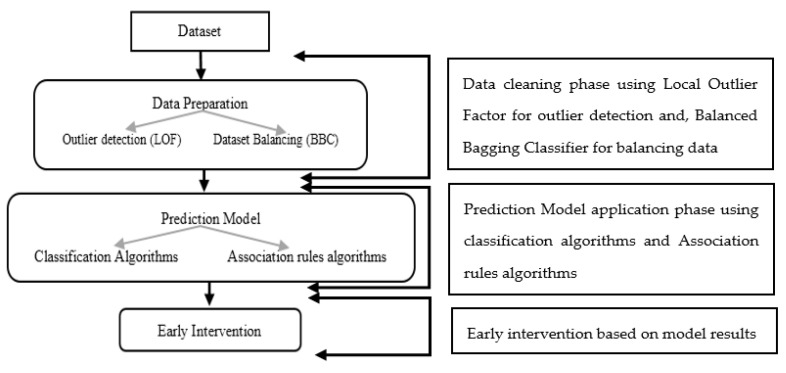
Proposed Integrative Approach Framework.

This section explains the details of the proposed integrative approach for diabetes prediction. The approach starts with a cleaned dataset (LOF), and the BBC is applied to solve the imbalanced dataset problem. Finally, classification algorithms are combined with association rules to predict diabetes. Figure 1 illustrates the proposed approach with its different steps.

The following subsections present the dataset, the LOF, the BBC, and the details of the applied classification algorithms and association rules.

### 3.1. Data Preparation

We used a dataset from the University of California machine learning repository (UCI) [41]. The dataset was collected using direct questionnaires from patients of the Sylhet Diabetes Hospital in Sylhet, Bangladesh. It contains 520 instances (320 positive and 200 negatives), 16 attributes (15 nominal and one numerical) for each patient, and one class attribute to classify patients as early-stage diabetics or not. Table 1 shows the dataset characteristics.

**Figure 2:**
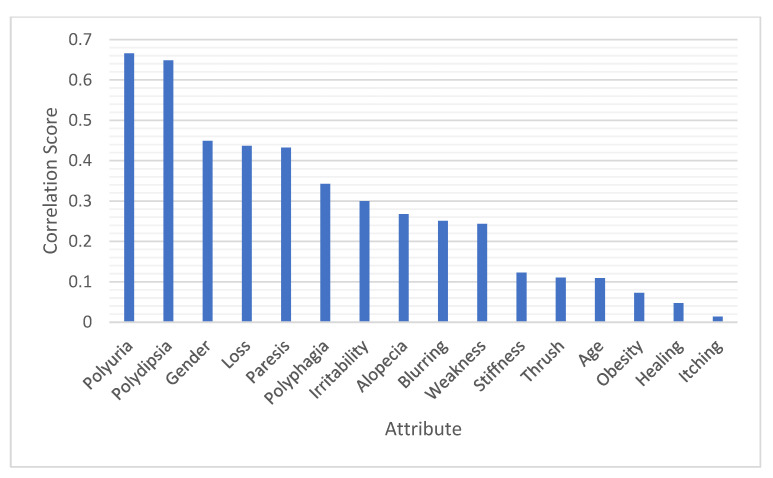
Attribute scores from the correlation technique.

**Figure 3:**
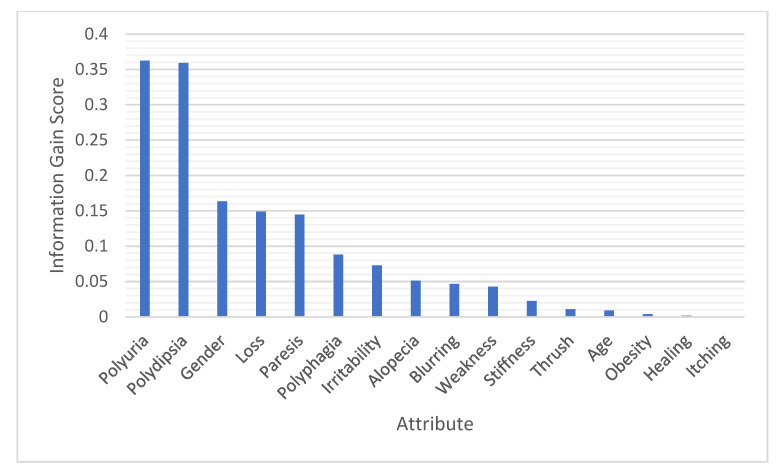
Attribute scores from the information gain technique.

Data preprocessing was applied to the raw data to handle missing values and choose the most relevant attributes for further investigation. Attributes selected for further investigation were polyuria, polydipsia, gender, sudden weight loss, partial paresis, polyphagia, irritability, alopecia, visual blurring, weakness, age, and muscle stiffness, while genital thrush, obesity, delayed healing, and itching were ignored due to their relatively low scores. Figure 2 and Figure 3 represent the correlation score (C) and information gain score (I.G.) for each attribute, respectively, using WEKA [42].

### 3.2. Outlier Detection (LOF)

In this study, LOF was used as an outlier detection technique. Outlier detection can be divided into two types, global outlier detection and local outlier detection. All data points are considered in global outlier detection, and a data point is treated as an outlier if it is too far from all other data points [43]. In local outlier detection, only a subset of the data is considered. Local outlier detection is based on comparing each data point and its neighbourhood, measured using the k-Nearest Neighbour (KNN) algorithm [44].

LOF is defined by using density-based techniques [33]. First, the k-distance of data point a is calculated using the Euclidean n-dimensional space.

As in Equation (1), the outlying degree should be first calculated to find the LOF for each data point and the LOF for a data point; four calculations must be done first [33].

(1) $$d\left(a,b\right)=\sqrt{\sum_{i=1}^{n}{\left(a_{i}-b_{i}\right)}^{2}}$$

where a is a data point in dataset d, b is the farthest neighbor from a, and k is any positive integer satisfying the following conditions:

- At least there are *k* data points b′∈D\{a}, satisfying that d(a,b′)≤d(a,b);
- At most, there are *k* − 1 data points b′∈D\{a}, satisfying that d(a,b′)<d(a,b);

Second, the k-nearest neighbours of a are calculated using Equation (2):

(2) $$N_{k-distance\left(a\right)}\left(a\right)=\left\{c\;\in D\backslash \left\{a\right\}|d\left(a,c\right)\leq k-distance\;\left(a\right)\right\}$$

where c is  any data point whose distance to a is smaller than the k-distance (a).

Third, the reachability distance of a concerning b is calculated using Equation (3):

(3) $$reach-distance_{k}\left(a,b\right)=max\left\{k-distance\;\left(b\right)d\left(a,b\right)\right\}$$

where *k* is any positive integer.

Fourth, the local reachability distance of a is calculated using Equation (4):

(4) LrdMinPts(a)=1(∑b∈NMinPts(a)reach−distanceMinPts(a,b)MinPts(a))

where MinPts is the minimum number of nearest neighbours of a. Finally, the LOF is calculated using Equation (5):

(5) $$LOF_{MinPts}\left(a\right)=\frac{\sum_{n\in N_{MinPts\left(a\right)}}\frac{lrd_{MinPts}\left(b\right)}{lrd_{MinPts}\left(a\right)}}{\left|N_{MinPts\left(a\right)}\right|}$$

Once an LOF score is calculated for each data point in the data set, a threshold θ score is used to determine if the point is an outlier or not. After applying the LOF to our data set, 30 data points were removed as recognized as outliers, and only 490 data points were left for further investigation.

### 3.3. Imbalanced Dataset Handling (BBC)

The basic idea of imbalanced data bagging is to redistribute the data points from minority and majority classes in bootstraps [38]. Data point redistribution can be handled in different ways. However, it has been shown that under-sampling, i.e., decreasing the majority class, outperforms over-sampling, i.e., increasing the size of the minority class [38,39]. This study applied an active balancing bagging algorithm (ABB) proposed by Błaszczyński & Stefanowski to balance our dataset [38].

The applied ABB is based on two main algorithms: the Exactly Balanced Bagging Algorithm (EBB) and the Neighborhood Balanced Bagging Algorithm (NBB). Before applying the ABB, our data set had a majority class of 320 data points and a minority class of 200 data points. After applying the ABB algorithm, the dataset was balanced to 260 for the majority and minority classes, respectively. It is believed that building a prediction model based on balanced data would lead to more accurate results.

### 3.4. Prediction Model

After outlier detection and data balancing, the prediction model was applied to the cleaned dataset. We implemented the prediction model using the Weka platform [40]. Four classification algorithms and association rules were used to extract knowledge that helps predict diabetes in its early stage. The following subsections describe the applied algorithms in detail.

#### 3.4.1. Artificial Neural Networks (ANNs)

**Figure 4:**
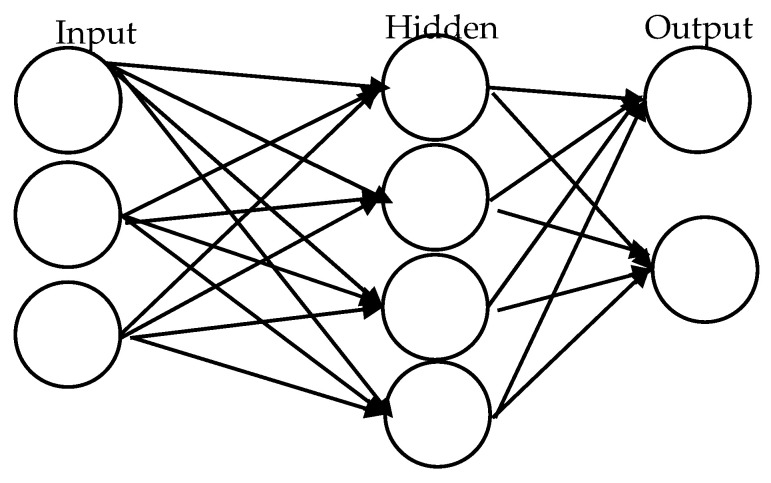
Artificial Neural Network Architecture.

An artificial neural network (ANN) is the part of a computer system that imitates the human brain. It is the backbone of artificial intelligence as it can solve problems that normal humans sometimes cannot solve. ANNs have self-learning capabilities to produce better results if data are available. As described in Figure 4, the ANNs are connected nodes responsible for processing and transmitting information to and from the brain. ANNs have been applied in many areas such as optimization problems, E-mail services, E-commerce, pattern recognition, clustering and categorization, prediction and forecasting, and deep learning techniques. The ANNs’ ability to learn automatically from examples makes them more attractive than other artificial intelligence techniques.

#### 3.4.2. Decision Trees (DTs)

**Figure 5:**
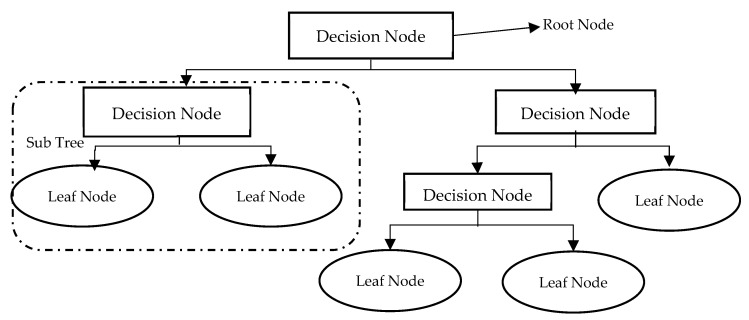
Decision Trees Architecture.

Decision Trees (DTs) are one of the most popular classification techniques. As shown in Figure 5, DTs are simply flowcharts that take the structure of an upside-down tree. Tree nodes represent tests, tree branches represent test results, and tree leaves repre-sent class labels. The DT classifier is very suitable for preparatory knowledge discovery as its construction does not need domain knowledge. A DT has many noteworthy characteristics which gives it superiority over other classifying techniques. First, it can handle large amounts of complex structured data. Second, its classifier has a high level of accuracy as its induction can learn knowledge during the classification process. Third, the DT also has its own generated rules for prediction, which makes model in-terpretation easier and more obvious. Last but not least, DTs have been widely used recently as data mining and machine learning techniques for predicting system be-haviour, making them very suitable for predicting early-stage diabetes.

#### 3.4.3. Support Vector Machines (SVMs)

**Figure 6:**
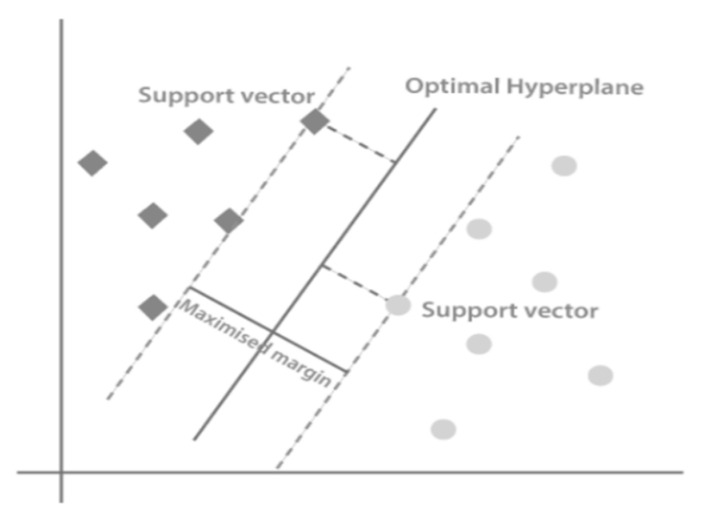
Support vector machine algorithm.

A Support Vector Machine (SVM) is a supervised machine learning algorithm for regression and classification models. As represented in Figure 6, in the SVM algorithm, each feature value is represented on an n-dimensional space by a specific coordinate value. In contrast, every data item is expressed by an element in the feature space. Classification is then done by determining the hyper-plane that best distinguishes the two classes. The SVM classifier separates the two classes in the best possible way and finds the optimal hyperplane by applying the concept of margin [45]. The SVM classifier can be applied to both non-linear and linear datasets. Kernel functions can transform non-linear data sets into linear datasets [46]. The kernel functions adopted in this study are radial basis, sigmoid, and polynomial functions.

#### 3.4.4. K-Nearest Neighbor (KNN)

**Figure 7:**
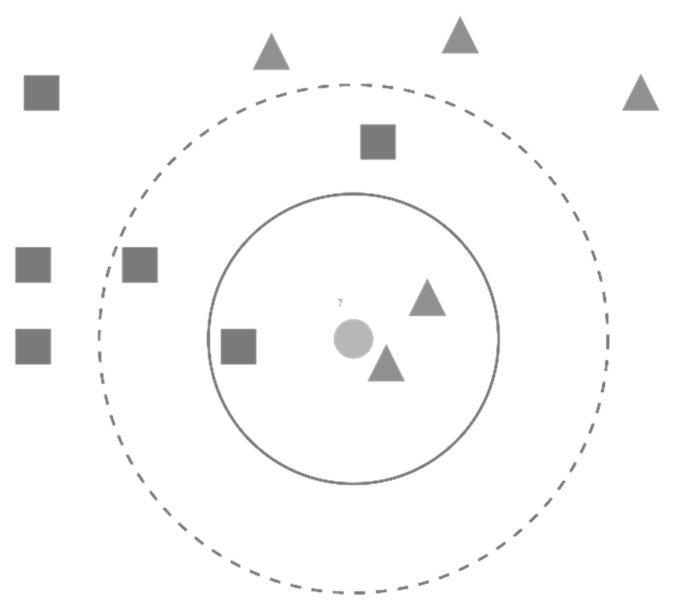
K-Nearest Neighbor Algorithm.

KNN is widely used in dataset classification as an immediate-based learning method. As shown in Figure 7, data points are represented in an n-dimensional space. Given a new data point, KNN uses similarity tests to determine how similar the new data entry is to the data points of the dataset to classify it accordingly. KNN determines the new data point class by estimating the K closest points to the new one, where the KNN employs majority voting to estimate the class of the new data point. Classes of new data points are found based on similar neighbors. The similarity between neighbors is commonly determined by the Euclidian distance between two neighbors, as presented in Equation (6):

(6) $$\;\;\;\;\;Dist\left(a,b\right)=\sqrt{\underset{i}{\sum }{\left(a_{i}-b_{i}\right)}^{2}}$$

a and b are any two data points. They are considered similar if their Euclidian distance is short.

#### 3.4.5. Association Rule

The problem of finding association rules from the dataset is known in the literature as the market basket problem [21]. The market basket problem is defined as a set of items in different baskets, and one should find how different items in different baskets are related. An a priori algorithm is one of the most commonly used association rule algorithms [21]. The algorithm adopts a level-based search, where n+1 item-sets are found based on n item-sets [47]. Recurrent item-sets are expanded in the candidate generation, where candidates are tested against the dataset already obtained, and association rules are estimated accordingly. The breadth-first algorithm and hash tree structure are the main tools utilized in the a priori algorithm. Extracted association rules are very helpful in predicting class values for early-stage diabetes. However, different metrics may measure how strong the extracted rule is. Some of these metrics are described below.

- Support is the percentage of transactions in the dataset containing item sets a and b. The support of an association rule a→b is given by:
  Support (a→b) = Support (a∪b)= P(a∪b) [52, 24].
- Confidence is the percentage of transactions in the dataset with item-set a containing the item-set *b*. The confidence equation is given by:
  $$\mathrm{Confidence}\;(a\to b)\;=\;P(b|a)\;=\;\frac{Support\left(a\cup b\right)}{Support\left(a\right)}\;[21,\;47].$$
- Lift is used to measure how often a and b occur together if both are statistically independent [47]. The lift of rule a→b is given by:
  $$lift\left(a\to b\right)\;=\;\frac{Confidence}{Expected\;Confidence}\;=\;\frac{Confidence\;\left(a\to b\right)}{Support\left(b\right)}\;$$
- Conviction measures the implication strength of the rule from statistical independence [47]. Conviction is given by:
  $$Conviction\left(a\to b\right)=\frac{1-Support\left(b\right)}{1-Confidence\left(a\to b\right)}$$

After applying different classification algorithms, our model results were compared with the testing dataset to ensure model accuracy. We used performance measures to assess the performance of the prediction model.

The following subsection describes the performance measures used in this study.

### 3.5. Performance Measures

A confusion matrix was generated, as shown in Table 2, where T.P. (True Positive) and T.N. (True Negative) are correctly classified outcomes, while F.P. (False Positive) and F.N. (False Negative) are incorrectly classified outcomes.

In order to measure our model accuracy four different performance measures were utilized, namely:

- Accuracy = (TP + TN)/(TP + FP +TN + FN);
- Sensitivity = TP/(TP + FN);
- Specificity = TN/(FP + TN);
- Precision = TP/(TP + FP).

## 4. Experimental Results

This study four classification algorithms and association rules to the dataset for predicting diabetes. Ten-fold cross-validation was used for dividing the dataset into training and testing data. Performance measures for each classification algorithm are shown in Table 3.

As represented in Table 3, KNN recorded the highest accuracy (97.36%) among other classifiers. That level of accuracy was achieved when the *k-parameter* was set to 1, as represented in Table 4, which describes the KNN algorithm accuracy for different K-values. The K value describes the count of the nearest neighbours. K is the number of neighbours “voting” on the test class. For example, if k = 1, test examples are given the same caption as the closest example in the training set. If k = 3, the captions of the three closest classes are checked, the most common label is assigned, and so on for larger Ks.

We applied an a priori algorithm to extract Lift matrix-based strong rules. Based on the correlation score (C) and information gain score (I.G.) for each attribute, certain attributes selected for further investigation were polyuria (P.u), polydipsia (P.d), gender (Gen), sudden weight loss (SWL), partial paresis (P.P.), polyphagia (P.ph), irritability (Irr), alopecia (Alo), visual blurring (V.B.), weakness (W), age, and muscle stiffness (M.S.). In addition, rule a determines b was defined, where a contains selected attributes set and *b* contains the class early-stage diabetics or not.

Four strong rules were generated using the a priori algorithm:

- Rule 1: {P.u: yes, P.d: yes, P.ph: yes, Alo: yes, W:yes, SWL: yes}=={class: diabetics};
- Rule 2: {P.u: yes, P.ph: yes, Alo: yes, W: yes, SWL: yes, MS: yes }=={class: diabetics};
- Rule 3: {P.u: yes, P.d: yes, P.ph: yes, Irr: yes, W: yes, SWL: yes }=={class: diabetics};
- Rule 4: {P.u: no, P.d: no, P.ph: no, SWL: no} == {class: not-diabetics}.

The strength of each rule is represented in Table 5. Confidence ranges from 0 to 1; where 0 indicates no reliance, and 1 indicates high reliability. Lift predicts the performance of an association rule as a response enhancer. It ranges from 0 to ∞, where a positive lift smaller than 1 indicates negative interdependence between a and b. In contrast, if the Lift is greater than 1, then a and b are positively interdependent. If lift = 1, then a and b appear together frequently, assuming conditional independence. Confidence and lift limitations are addressed using conviction. Conviction evaluates the rule’s implication degree. It ranges from 0.5 to ∞, where if conviction = 1, the rule is independent, and items are not associated. If conviction > 1, rules are interesting, and the higher the value, the more relationship between items.

## 5. Discussion

We developed a prediction system for predicting diabetes in its early stage. Four classification algorithms were utilized in addition to an a priori algorithm that discovered relationships between various factors. The Local Outlier Factor (LOF)-based outlier detection technique detected outlier data. A Balanced Bagging Classifier (BBC) technique was used to balance data distribution. Finally, integration between association rules and classification algorithms was used to develop a prediction model based on real data. Performance results showed that the proposed integrative approach significantly enhanced model accuracy compared with other models and previous study results, achieving a prediction accuracy of 97.36%.

The results showed that KNN outperformed other classification algorithms in predicting diabetes. It was shown that the accuracy of the KNN algorithm was negatively related to the increasing values of K. In terms of sensitivity and specificity, SVM achieved slightly better results than those recorded by the KNN algorithm. However, in terms of precision, KNN had superiority, followed by DT, which recorded the least prediction accuracy per our results.

We applied the a priori algorithm to 12 attributes to extract Lift matrix-based association rules. Four main attributes, namely, polyuria (P.u), polydipsia (P.d), polyphagia (P.ph), and sudden weight loss (SWL), recorded the highest ranks of correlation with prediction class attributes. Thus, most rules were extracted from these attributes. When polyuria, polydipsia, polyphagia, and sudden weight loss values are yes, the class value is diabetics. In contrast, if the values of those attributes are set to no, then the class value is not diabetics.

## 6. Conclusions

A diabetes prediction system in its early stage is proposed. A real dataset was used to predict patient cases. Dataset preprocessing was conducted to produce relevant and more consistent data. Four algorithms were applied: ANN, DT, SVM, and KNN for data classification. Results revealed that KNN had the highest accuracy of 97.36% among the other applied algorithms. An a priori algorithm extracted association rules based on the Lift matrix. Four association rules from 12 attributes with the highest correlation and information gain scores relative to the class attribute were produced.

Early prediction of diabetics is very significant for critical health-related decision-making, patients’ health, and overall healthcare costs in the healthcare systems. This study has many practical implications. It provides an efficient prediction system with high accuracy for diabetic patients. With early diagnosis and intervention, it is possible to slow disease progression and prevent future health complications.

One of the study limitations is that the proposed approach cannot be easily generalized to other classification methods, as it does not deal ideally with continuous data types. Another limitation is that the approach may not work properly on large datasets. Future studies will include extending the proposed approach by applying different classifiers and association rules generating algorithms.

Future studies will also investigate different parameters for the proposed prediction system and its potential application to other medical datasets. Furthermore, outliers can be handled by different data balancing algorithms.

## Figures and Tables

**Table 1:** Dataset attribute types and missing values.

Attributes (520 Instances)		
Name	Type	Missing Values (%)
Age	Numerical	2
Gender (Gen)	Nominal	1
Polyuria (P.u)	Nominal	1
Polydispsia (P.d)	Nominal	2
Sudden Weight Loss (SWL)	Nominal	3
Weakness (W)	Nominal	3
Polyphagia (P.ph)	Nominal	4
Genital Thrush (GT)	Nominal	3
Visual Blurring (VB)	Nominal	3
Itching (I)	Nominal	4
Irritability (Irr)	Nominal	5
Delayed healing (DH)	Nominal	4
Partial paresis (PP)	Nominal	5
Muscle stiffness (MS)	Nominal	6
Alopecia (Alo)	Nominal	4
Obesity (Obs)	Nominal	7

**Table 2:** Confusion matrix.

Predicted Data		
Real Data	Positive	Negative
Positive	TP	FN
Negative	FP	TN

**Table 3:** Performance measures for different classifiers.

	Accuracy%	Sensitivity%	Specificity%	Precision%
ANN	96.89	98.76	95.43	96.90
DT	95.78	97.56	93.87	97.44
DT (with no LOF)	95.77	97.59	93.78	97.34
SVM	96.22	99.87	95.98	96.89
KNN	97.36	99.21	95.94	98.22

**Table 4:** KNN accuracy for different K-values.

**Accuracy%**	97.36	96.98	96.86	96.56
**K-Value**	1	3	5	7

**Table 5:** The strength of each rule.

	Confidence %	Lift	Conviction
Rule1	89	2.20	3.24
Rule2	84	1.34	2.89
Rule3	88	1.89	2.96
Rule4	92	2.34	4.34

## Data Availability

All data are available in the manuscript.

## References

[1] World Health Organization (2019). Non-Communicable Diseases. https://www.who.int/en/news-room/fact-sheets/detail/noncommunicable-diseases.

[2] World Health Organization (2016). NCD Mortality and Morbidity. https://www.who.int/gho/ncd/mortality_morbidity/en.

[3] World Health Organization (2016). Projections of Mortality and Causes of Death, 2016 to 2060. https://www.who.int/healthinfo/global_burden_disease/projections/en.

[4] Sonawane J.S., Patil D.R. Prediction of heart disease using multilayer perceptron neural network. Proceedings of the International Conference on Information Communication and Embedded Systems (ICICES2014).

[5] Veena V.V., Anjali C. Prediction and diagnosis of diabetes mellitus—A machine learning approach. Proceedings of the 2015 IEEE Recent Advances in Intelligent Computational Systems (RAICS).

[6] Wild S., Roglic G., Green A., Sicree R., King H. (2004). Global prevalence of diabetes: Estimates for the year 2000 and projections for 2030. Diabetes Care.

[7] Rubino F. (2008). Is type 2 diabetes an operable intestinal disease? A provocative yet reasonable hypothesis. Diabetes Care.

[8] Wu H., Yang S., Huang Z., He J., Wang X. (2018). Type 2 diabetes mellitus prediction model based on data mining Inform. Med. Unlocked.

[9] Meng X.-H., Huang Y.-X., Rao D.-P., Zhang Q., Liu Q. (2013). Comparison of three data mining models for predicting diabetes or prediabetes by risk factors. Kaohsiung J. Med. Sci..

[10] Batista G.E.A.P.A., Prati R.C., Monard M.C. (2004). A study of the behavior of several methods for balancing machine learning training data. ACM SIGKDD Explor. Newsl..

[11] Chen T., Shi X., Wong Y.D. (2019). Key feature selection and risk prediction for lane-changing behaviors based on vehicles’ trajectory data. Accid. Anal. Prev..

[12] Domingues R., Filippone M., Michiardi P., Zouaoui J. (2018). A comparative evaluation of outlier detection algorithms: Experiments and analyses. Pattern Recognit..

[13] Calheiros R.N., Ramamohanarao K., Buyya R., Leckie C., Versteeg S. (2017). On the effectiveness of isolation-based anomaly detection in cloud data centers. Concurr. Comput. Pract. Exper..

[14] Yan H., Jiang Y., Zheng J., Peng C., Li Q. (2006). A multilayer perceptron-based medical decision support system for heart disease diagnosis. Expert Syst. Appl..

[15] Aqlan F., Markle R., Shamsan A. Data mining for chronic kidney disease prediction. Proceedings of the IIE Annual Conference. Institute of Industrial and Systems Engineers (IISE).

[16] Arasu D., Thirumalaiselvi R. (2017). Review of chronic kidney disease based on data mining techniques. Int. J. Appl. Eng. Res..

[17] Tun N.N., Arunagirinathan G., Munshi S.K., Pappachan J.M. (2017). Diabetes mellitus and stroke: A clinical update. World J. Diabetes.

[18] World Health Organization World Health Statistics 2012. https://www.who.int/gho/publications/world_health_statistics/2012/en.

[19] Alloubani A., Saleh A., Abdelhafiz I. (2018). Hypertension and diabetes mellitus as a predictive risk factor for stroke. Diabetes Metab. Syndr. Clin. Res. Rev..

[20] Brossette S.E., Sprague A.P., Hardin J.M., Waites K.B., Jones W.T., Moser S.A. (1998). Association rules and data mining in hospital infection control and public health surveillance. J. Am. Med. Inform. Assoc..

[21] Agrawal R., Imielinski T., Swami A. (1993). Database Mining: A Performance Perspective. IEEE Trans. Knowl. Data Eng..

[22] Agrawal R., Imielinski T., Swami A. Mining association rules between sets of items in large databases. Proceedings of the 1993 ACM SIGMOD International Conference on Management of Data.

[23] Pendyala S., Fang Y., Holliday J., Zalzala A. A text mining approach to automated healthcare for the masses. Proceedings of the IEEE Global Humanitarian Technology Conference (GHTC 2014).

[24] Tsanas A., Little M.A., Mcsharry P.E., Spielman J., Ramig L.O. (2012). Novel speech signal processing algorithms for high-accuracy classification of Parkinson’s disease. IEEE Trans. Biomed. Eng..

[25] Otunaiya K., Muhammad G. (2019). Performance of data mining techniques in predicting chronic kidney disease. Comput. Sci. Inf. Technol..

[26] Yu W., Liu T., Valdez R., Gwinn M., Khoury M.J. (2010). Application of support vector machine modeling for prediction of common diseases: The case of diabetes and prediabetes. BMC Med. Inform. Decis. Mak..

[27] Ozcift A., Gulten A. (2011). Classier ensemble construction with rotation forest to improve medical diagnosis performance of machine learning algorithms. Comput. Methods Programs Biomed..

[28] Chen W., Chen S., Zhang H., Wu T.A. A hybrid prediction model for type 2 diabetes using K-means and decision tree. Proceedings of the 2017 8th IEEE International Conference on Software Engineering and Service Science (ICSESS).

[29] Saragih T.H., Fajri D.M.N., Mahmudy W.F., Abadi A.L., Anggodo Y.P. (2018). Jatropha curcas disease identification with extreme learning machine. Indones. J. Electr. Eng. Comput. Sci..

[30] Rahmi A., Wijayaningrum V.N., Mahmudy W.F., Parewe A.M.A.K. (2016). Offline signature recognition using back propagation neural network. Indones. J. Electr. Eng. Comput. Sci..

[31] Gangadharrao M.S., Lahiri K. (1992). Introduction to Econometrics.

[32] Syafrudin M., Fitriyani N., Alan G., Rhee J. (2018). An affordable, fast early warning system for edge computing in assembly line. Appl. Sci..

[33] Alghushairy O., Alsini R., Soule T., Ma X. (2020). A review of local outlier factor algorithms for outlier detection in big data streams. Big Data Cogn. Comput..

[34] Yan K., You X., Ji X., Yin G., Yang F. A Hybrid Outlier Detection Method for Health Care Big Data. Proceedings of the 2016 IEEE International Conferences on Big Data and Cloud Computing (BDCloud), Social Computing and Networking (SocialCom), Sustainable Computing and Communications (SustainCom) (BDCloud-SocialCom-SustainCom).

[35] Budiarto E.H., Permanasari A.E., Fauziati S. Unsupervised anomaly detection using K-means, local outlier factor, and one class SVM In Proceedings of the 2019 5th International Conference on Science and Technology (ICST).

[36] Farquad M.A.H., Bose I. (2012). Preprocessing unbalanced data using support vector machine. Decis. Support Syst..

[37] Harliman R., Uchida K. (2018). Data- and algorithm-hybrid approach for imbalanced data problems in deep neural network. Int. J. Mach. Learn. Comput..

[38] Błaszczyński J., Stefanowski J. (2017). Actively balanced bagging for imbalanced data. International Symposium on Methodologies for Intelligent Systems.

[39] Anbarasi M.S., Janani V. Ensemble classifier with Random Forest algorithm to deal with imbalanced healthcare data. Proceedings of the 2017 International Conference on Information Communication and Embedded Systems (ICICES).

[40] Tuli S., Basumatary N., Gill S.S., Kahani M., Arya R.C., Wander G.S., Buyya R. (2020). HealthFog: An ensemble deep learning based Smart Healthcare System for Automatic Diagnosis of Heart Diseases in integrated IoT and fog computing environments. Future Gener. Comput. Syst..

[41] Karthick K. Early Stage Diabetes Risk Prediction Dataset. https://www.cs.waikato.ac.nz/ml/weka/.

[42] (2019). Weka 3: Data Mining Software in Java. https://www.cs.waikato.ac.nz/ml/weka/.

[43] Knox E.M., Ng R.T. Algorithms for mining distance-based outliers in large datasets. Proceedings of the International Conference on Very Large Data Bases.

[44] Souiden I., Brahmi Z., Toumi H. (2017). A Survey on Outlier Detection in the Context of Stream Mining: Review of Existing Approaches and Recommendations. Intelligent Systems Design and Applications.

[45] Zeynu S., Patil S. (2018). Survey on prediction of chronic kidney disease using data mining classification techniques and feature selection. Int. J. Pure Appl. Math..

[46] Han J., Kamber M., Pei J. (2011). Data mining concepts and techniques third edition. Morgan Kaufmann Ser. Data Manage. Syst..

[47] Brijs T., Vanhoof K., Wets G. (2003). Defining interestingness for association rules. Int. J. Inf. Theor. Appl..

